# Supplementary material for: Sphingomyelin regulates the transcriptional machinery in nuclear lipid microdomains
Source: Commun Biol. 2025 Aug 29;8:1303. doi: 10.1038/s42003-025-08697-2 (PMC12397320; doi:10.1038/s42003-025-08697-2)
Supplement: Supplementary file 2 — Supplementary Information [file 42003_2025_8697_MOESM2_ESM.pdf]

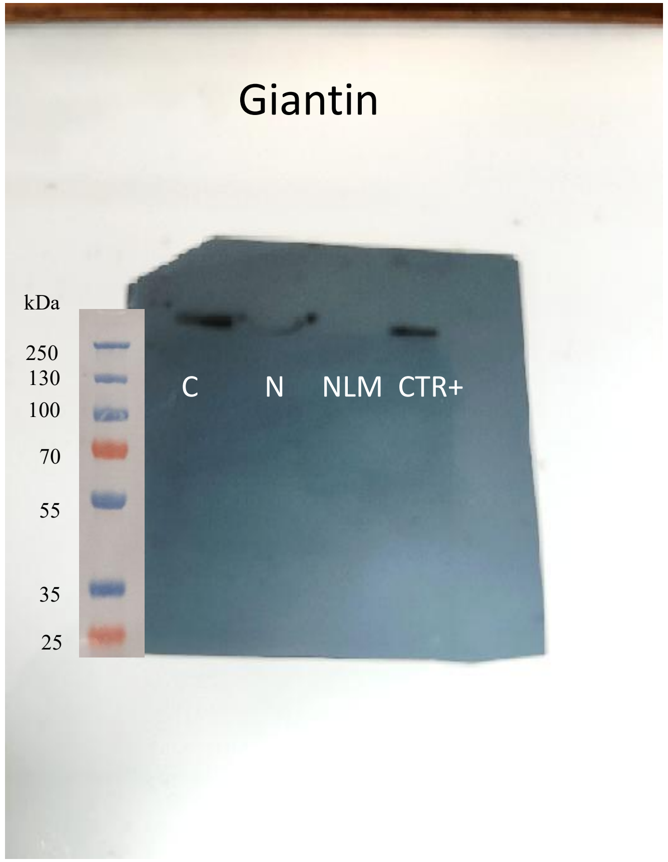

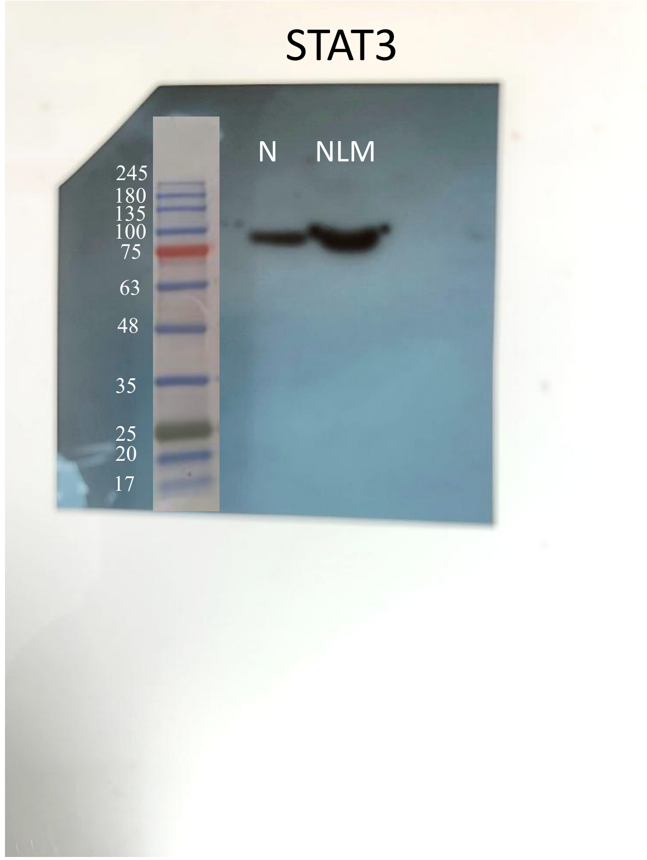

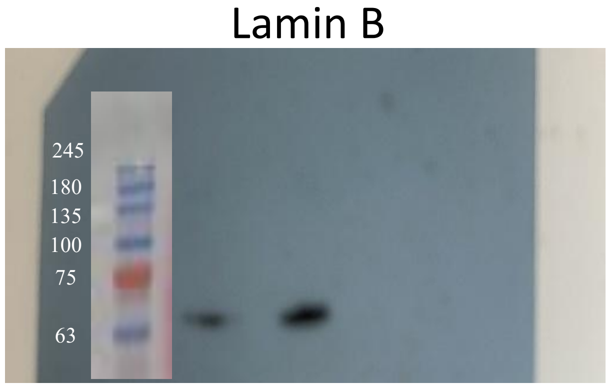

Supplementary Figure 1. Original Western Blotting

RNAseq

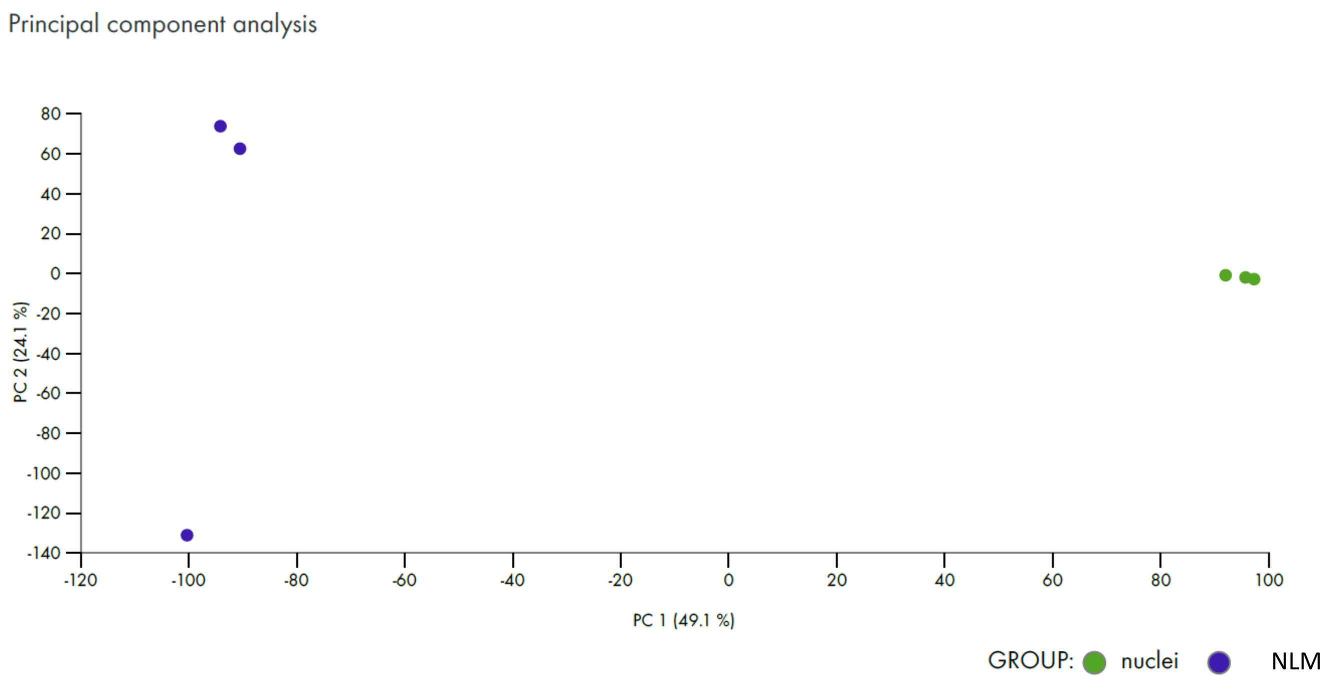

Supplementary Figure 2. Principal component analysis (PCA) performed on the expression profiles of the nuclei and nuclear lipid microdomains (NLM). The two first principal components are plotted, explaining together 82% of the observed variance, and each experimental condition is highlighted by a different color. The samples form two separate and homogeneous clusters, confirming that the two experimental conditions represent distinct samples.

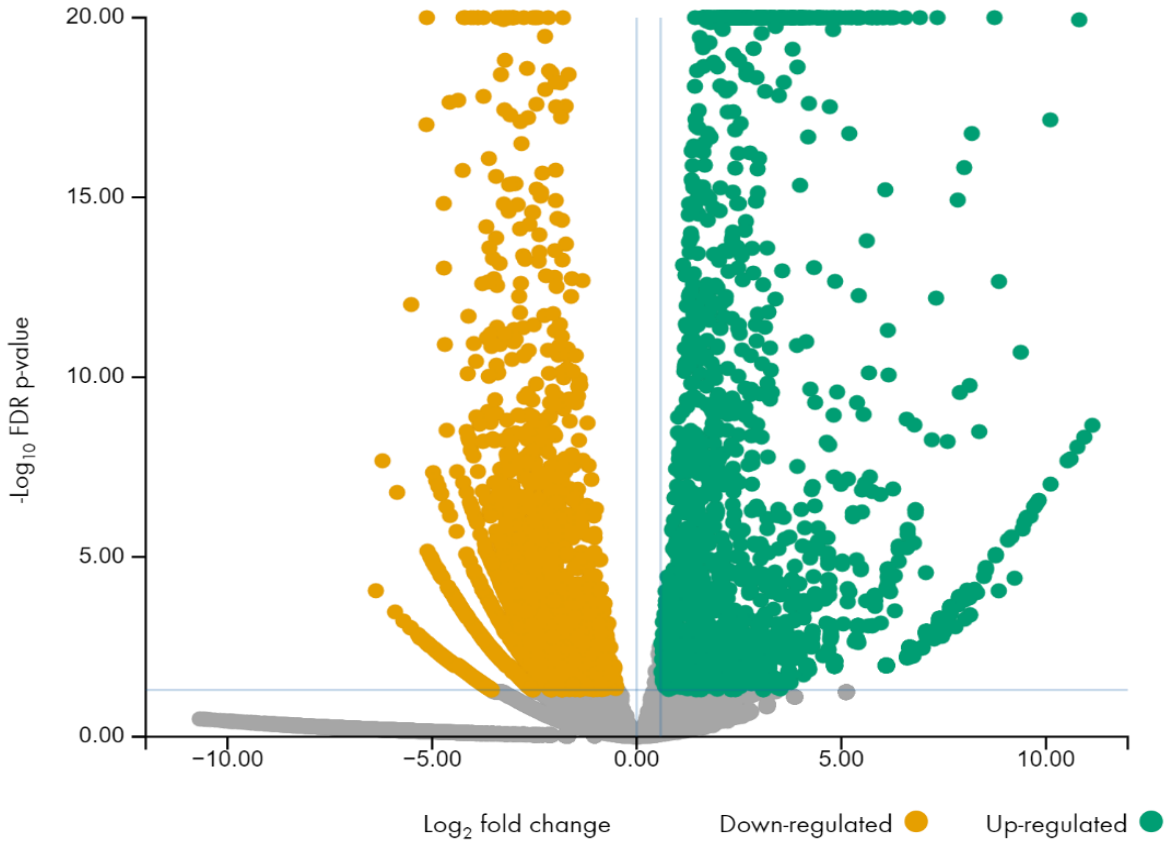

Supplementary Figure 3: VOLCANO PLOT. up- and down-regulated mRNA in nuclear lipid microdomains (NLMs) compared to nuclei

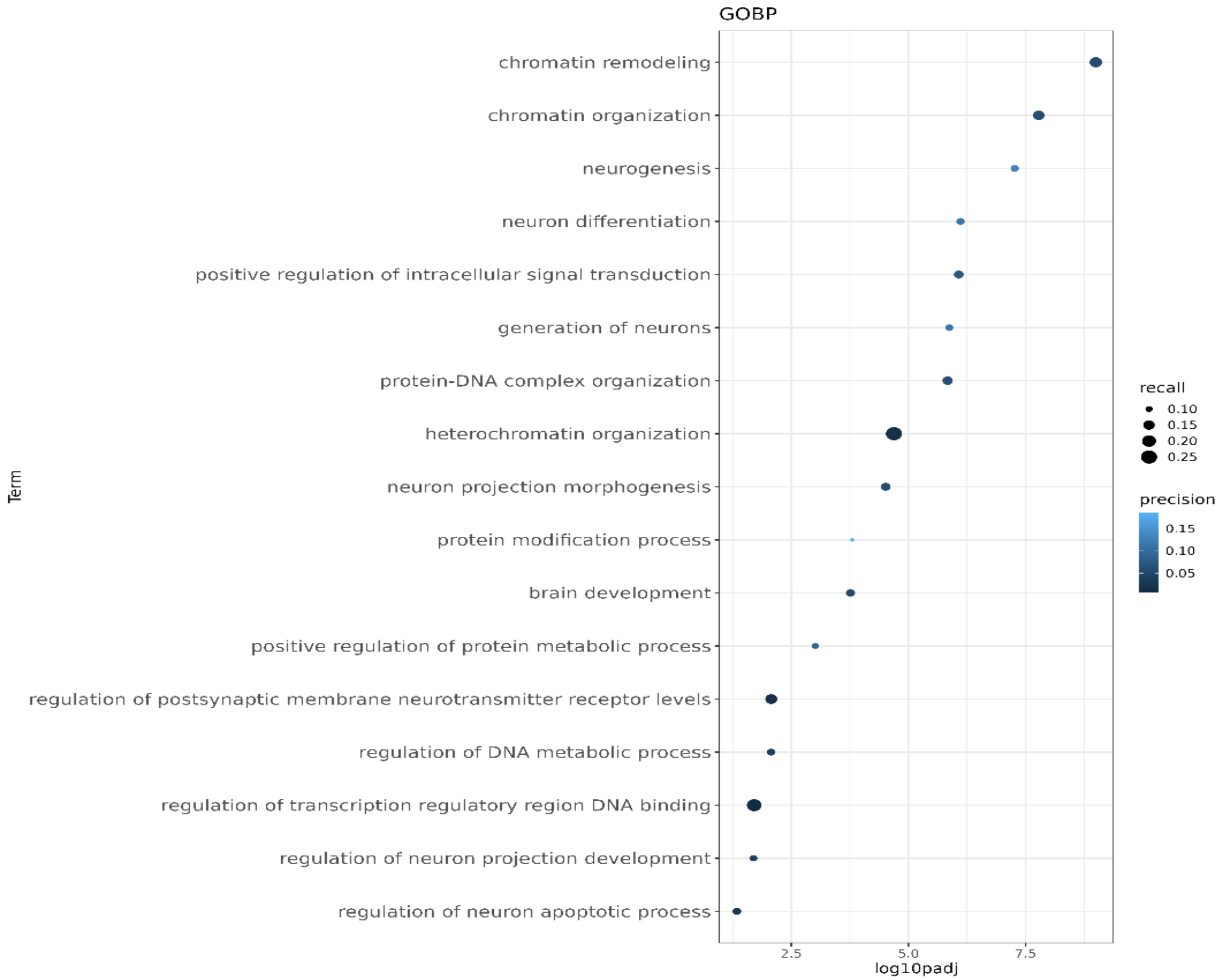

Supplementary Figure 4. Enrichment analysis of highly concentrated mRNAs targeted by highly concentrated miRNA in NLMs. The x axis reports the -log10 (adjusted p-value) while the term names are reported on the y-axis. The color intensity represents the precision, defined as the proportion of genes in the input list that are annotated to the function (defined as intersection_size/query_size) while the circle dimension represents the recall, defined as the proportion of functionally annotated genes that the query recovers (defined as intersection_size/term_size).

miRNA seq

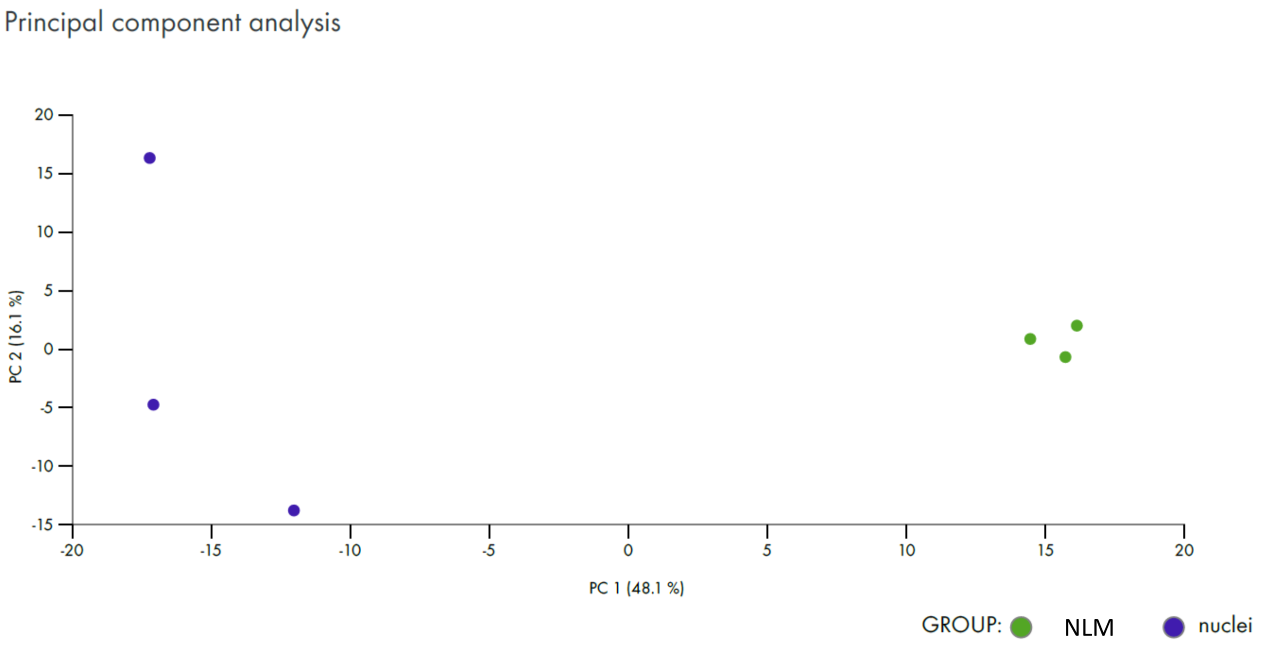

Supplementary Figure 5. Principal component analysis (PCA) performed on the expression profiles of the nuclei and intranuclear complex. The two first principal components are plotted, explaining together 82% of the observed variance, and each experimental condition is highlighted by a different color. The samples form two separate and homogeneous clusters, confirming that the two experimental conditions represent distinct samples.

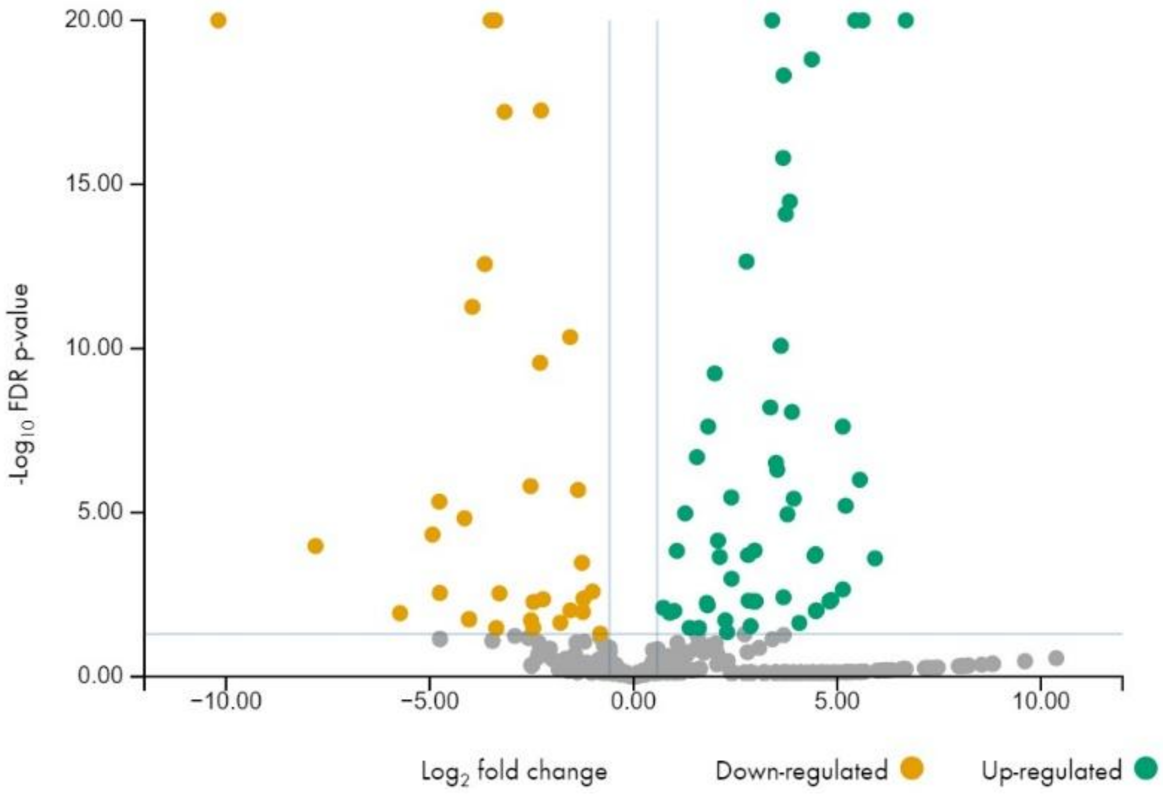

Supplementary Figure 6: VOLCANO PLOT. up- and down-regulated miRNA in nuclear lipid microdomains (NLMs) compared to nuclei

Canonical Pathways | Diseases and functions

Diseases and functions	Prediction	Function	z-score	Molecules	p-value	-log10 of p-value
Chronic epilepsy	-	chronic epilepsy	-	25	2.55e-38	37.593
Sporadic amyotrophic lateral sclerosis	-	sporadic amyotrophic lateral sclerosis	-	30	4.76e-38	37.322
Sporadic psychological disorder	-	sporadic psychological disorder	-	31	1.07e-36	35.971
Nonobstructive azoospermia	-	nonobstructive azoospermia	-	32	1.52e-36	35.818
Kainic acid-induced epileptic seizure	-	kainic acid-induced epileptic seizure	-	22	3.80e-36	35.420
Early stage invasive cervical squamou...	-	invasive cervical squamous cell carcin...	-	23	1.01e-34	33.996
Chronic phase pilocarpine-induced st...	-	pilocarpine-induced status epilepticus	-	21	1.47e-34	33.833

Supplementary Table 1: Relationship of miRNA present in high concentration in nuclear lipid microdomains and diseases

chrom	strand	exonCount	exonSize	name	geneName	index exon or intron	left intron	right intron
chrX	-	2	570,90	circRNA	Zfx	4,3	chrX:94082299-94098282	chrX:94102380-94114195
chr12	-	2	74,104	circRNA	Strn3	3,2	chr12:51655483-51661150	chr12:51661713-51691421
chr11	-	1	2163	ciRNA	Recql5	2	chr11:115928486-115930676	
chr19	+	3	99,56,82	circRNA	Trub1	2,3,4	chr19:57453196-57458089	chr19:57472780-57483580
chr1	+	5	129,61,63,46,164	circRNA	Ccnt2	3,4,5,6,7	chr1:127775182-127791604	chr1:127799553-127801605
chr16	+	3	136,208,178	circRNA	Pkp2	4,5,6	chr16:16226291-16230640	chr16:16247043-16260325
chr18	+	2	107,196	circRNA	Ctnna1	2,3	chr18:35118981-35152600	chr18:35154540-35174252
chr6	-	2	76,233	circRNA	Cyren	3,2	chr6:34874695-34875536	chr6:34876771-34877574
chr5	+	4	86,84,50,70	circRNA	Depdc5	5,6,7,8	chr5:32868867-32875281	chr5:32887048-32893374
chr4	-	5	119,99,51,109,39	circRNA	Slc24a2	7,6,5,4,3	chr4:87011772-87028245	chr4:87076157-87226882
chr2	+	2	102,96	circRNA	Cstf3	2,3	chr2:104590702-104608870	chr2:104609186-104644288
chrX	-	1	161	ciRNA	Irak1	4	chrX:74022664-74022848	
chr11	+	2	47,250	circRNA	Acaca	2,3	chr11:84130130-84193078	chr11:84195687-84214157
chr10	+	2	98,179	circRNA	Ascc1	4,5	chr10:60007826-60012461	chr10:60013774-60025722
chr15	-	1	150	ciRNA	Kmt2d	2	chr15:98855898-98856068	
chr14	+	1	730	circRNA	Ercc6	5	chr14:32521059-32526148	chr14:32526878-32541225
chr19	+	3	99,56,82	circRNA	Trub1	2,3,4	chr19:57453196-57458089	chr19:57472780-57483580
chr6	+	2	133,121	circRNA	Ppp1r9a	3,4	chr6:4906844-5045935	chr6:5057578-5063990
chr6	+	2	99,206	circRNA	Ppfibp1	3,4	chr6:146938989-146976603	chr6:146978249-146990513
chr4	+	5	150,123,229,125,70	circRNA	Anp32b	2,3,4,5,6	chr4:46451349-46460089	chr4:46471467-46472086
chr4	+	3	191,132,147	circRNA	Ccdc171	5,6,7	chr4:83549777-83554693	chr4:83580894-83601607

Supplementary Table 2: nuclear circRNA with intron and exons (EIciRNAs) and ciRNA consisting of only introns

	Forward primer	Reverse primer
**Ehmt2**	GGAGCCAACATCAATGCCGTAG	TAGACAGGTGGAGCCATCCTCT
**Chaf1a**	AGAGTGTGGCAAGCACCTTCCT	CTTGCTGCTCTCCACAATGACC
**Dnm2**	ATGCTGCCACTGGACAACCTCA	GCCAGTTCAATCTGTCGAAGGTC
**Slc24a2**	CACTCTGTGGATTGCCGTGTTC	GCAGTCTGGAACACTTGTGCCT
**Ccnt2**	GCAAAAGTGGAAGAGCAGGCTC	ACCAGTTCTTGAGTCTGCTGAAG
**Depdc5**	GACCCAGAACAAGGACTCCCTA	GCAACAGAAGCCTGGCACTACA
**Gapdh**	CATCACTGCCACCCAGAAGACTG	ATGCCAGTGAGCTTCCCGTTCAG
**ActB**	CATTGCTGACAGGATGCAGAAGG	TGCTGGAAGGTGGACAGTGAGG

Supplementary Table 3: Target genes
