# Supplementary material for: Sphingomyelin regulates the transcriptional machinery in nuclear lipid microdomains
Source: Commun Biol. 2025 Aug 29;8:1303. doi: 10.1038/s42003-025-08697-2 (PMC12397320; doi:10.1038/s42003-025-08697-2)
Supplement: Supplementary file 5 — Reporting Summary [file 42003_2025_8697_MOESM5_ESM.pdf]

nature portfolio

Corresponding author(s): [illegible]

Last updated by author(s): July 25, 2025

Reporting Summary

Nature Portfolio wishes to improve the reproducibility of the work that we publish. This form provides structure for consistency and transparency in reporting. For further information on Nature Portfolio policies, see our Editorial Policies and the Edltor!al Policy Checklist

Statistics

For all statistical analyses, confirm that the following items are present in the figure legend, table legend, main text, or Methods section.

n/a	Confirmed
	X The exact sample size (n) for each experimental group/condition, given as a discrete number and unit of measurement
	X A statement on whether measurements were taken from distinct samples or whether the same sample was measured repeatedly
	X The statistical test(s) used AND whether they are one- or two-sided<br>*Only common tests should be described solely by name, describe more complex techniques in the Methods section.*
X	A description of all covariates tested
X	A description of any assumptions or corrections, such as tests of normality and adjustment for multiple comparisons
	X A full description of the statistical parameters including central tendency (e.g. means) or other basic estimates (e.g. regression coefficient) AND variation (e.g. standard deviation) or associated estimates of uncertainty (e.g. confidence intervals)
X	For null hypothesis testing, the test statistic (e.g. *F*, I, r) with confidence intervals, effect sizes, degrees of freedom and P value noted<br>*Give P values as exact values whenever suitable.*
X	For Bayesian analysis, information on the choice of priors and Markov chain Monte Carlo settings
X	For hierarchical and complex designs, identification of the appropriate level for tests and full reporting of outcomes
X	Estimates of effect sizes (e.g. Cohen's *d*, Pearson's r), indicating how they were calculated

Our web collection on s his or hon tons articles on man a ffie ohms above.

Software and code

Policy information about availabiltty of computer code

Data collection	https://www.ncbi.nlm.nih.gov/geo/query/acc.cgi?acc=GSE262449
Data analysis	LipidBlast database, DESeq2, Tarbase, Gene Ontology database, Circexplorer2 software, STAR software

[illegible]

Data

Policy information about ava!labiltty of data

All manuscripts must include a data availability statement. This statement should provide the following information, where applicable:

- Accession codes, unique identifiers, or web links for publicly available datasets
- A description of any restrictions on data availability
- For clinical datasets or third party data, please ensure that the statement adheres to our policy

The datasets generated and/or analyzed during the current study are available in the NCBI GEO repository, link https://www.ncbi.nlm.nih.gov/geo/query/acc.cgi?acc=GSE262449

Research involving human participants, their data, or biological material

Policy information about studies with human participants or human data. See also policy Informat!on about sex, gender (identity/presentation), and sexual orientation and race, ethnicity and racism.

Reporting on sex and gender	NA
Reporting on race, ethnicity, or other socially relevant groupings	NA
Population characteristics	NA
Recruitment	NA
Ethics oversight	NA

Note that full information on the approval of the study protocol must also be provided in the manuscript.

Field-specific reporting

Please select the one below that is the best fit for your research. If you are not sure, read the appropriate sections before making your selection

X Life sciences ☐ Behavioural & social sciences ☐ Ecological, evolutionary & environmental sciences

For a reference copy of the document with all sections see nature.com/documents/nr-reporting-summary-flat.pdf

Life sciences study design

All stud res must d isclose on tfiese points even wfien the disco osu e is negative

Sample size	Three independent experiments of cell culture
Data exclusions	NA
Replication	Each experiment was performed in duplicate
Randomization	NA
Blinding	NA

Behavioural & social sciences study design

All stud res must d isclose on tfiese points even wfien the disco osu e is negative

Study description	
Research sample	

Sampling strategy

Data collection

Timing

Data exclusions

Non-participation

Randomization

Ecological, evolutionary & environmental sciences study design

All stud Yes must d!sclose on tfiese points even wfien the disc osu e !s negative

Study description

Research sample

Sampling strategy

Data collection

Timing and spatial scale

Data exclusions

Reproducibility

Randomization Descri were

Blinding

Did the study involve field work? keY NO

Field work, collection and transport

Field conditions

Location

Access & import/export

Disturbance

Reporting for specific materials, systems and methods

We require information from authors about some types of materials, experimental systems and methods used in many studies. Here, indicate whether each material, system or method listed is relevant to your study. If you are not sure if a list item applies to your research, read the appropriate section before selecting a response.

Materials & experimental systems

n/a	Involved in the study	
	X	Antibodies
	X	Eukaryotic cell lines
X		Palaeontology and archaeology
X		Animals and other organisms
X		Clinical data
X		Dual use research of concern
X		Plants

Methods

n/a	Involved in the study	
	X	ChIP-seq
X		Flow cytometry
X		MRI-based neuroimaging

Antibodies

Antibodies used	Anti-giantin, anti-STAT3, anti-lamin b
Validation	Manufacturing industry

Eukaryotic cell lines

Policy information about eell nes and Sex and Gender n Research

Cell line source(s)	Ninewells Hospital, Dundee, UK
Authentication	Ninewells Hospital, Dundee, UK
Mycoplasma contamination	Cells were negative for Mycoplasma contamination
Commonly misidentified lines (See ICLAC register)	

Palaeontology and Archaeology

Specimen provenance	
Specimen deposition	
Dating methods	

Tick this box to confirm that the raw and calibrated dates are available in the paper or in Supplementary Information.

Ethics oversight	

Note that full information on the approval of the study protocol must also be provided in the manuscript.

Animals and other research organisms

Policy information about stud involving animals; ARRIVE gu recommended for report!ng an mal research, and Sex and Gender in Research

Laboratory animals	

Wild animals [illegible]

Reporting on sex [illegible]

Field-collected samples [illegible]

Ethics oversight [illegible]

Note that full information on the approval of the study protocol must also be provided in the manuscript.

Clinical data

Policy information about clinical studies
All manuscripts should comply with the ICMJE ________ for publication of elimeal research and a completed CONSORT checklist must be included with all submi[illegible]

Clinical trial registration [illegible]

Study protocol [illegible]

Data collection [illegible]

Outcomes [illegible]

Dual use research of concern

Policy information about dual use research of concern

Hazards

Could the accidental, deliberate or reckless misuse of agents or technologies generated in the work, or the application of information presented in the manuscript, pose a threat to:

No	Yes	
X		Public health
X		National security
X		Crops and/or livestock
X		Ecosystems
	X	Any other significant area

Experiments of concern

Does the work involve any of these experiments of concern:

No	Yes	
X		Demonstrate how to render a vaccine ineffective
X		Confer resistance to therapeutically useful antibiotics or antiviral agents
X		Enhance the virulence of a pathogen or render a nonpathogen virulent
X		/ncrease transmissibility of a pathogen
X		Alter the host range of a pathogen
X		Enable evasion of diagnostic/detection modalities
X		Enable the weaponization of a biological agent or toxin
X		Any other potentially harmful combination of experiments and agents

Plants

Seed stocks

Novel plant genotypes

Authentication

ChIP-seq

Data deposition

X Confirm that both raw and final processed data have been deposited in a public database such as GEO.

Confirm that you have deposited or provided access to graph files (e.g. BED files) for the called peaks.

Data access links<br>*May remain private before publication.*	https://www.ncbi.nlm.nih.gov/geo/query/acc.cgi?acc=GSE262449
Files in database submission	mRNA, miRNA, cyrcRNA
Genome browser session<br>(e.g. UCSC)	

Methodology

Replicates

Sequencing depth

Antibodies

Peak calling parameters

Data quality

Software

Flow Cytometry

Confirm that:

The axis labels state the marker and fluorochrome used (e.g. CD4-FITC).

The axis scales are clearly visible. Include numbers along axes only for bottom left plot of group (a 'group' is an analysis of identical markers).

All plots are contour plots with outliers or pseudocolor plots.

A numerical value for number of cells or percentage (with statistics) is provided.

Methodology

Sample preparation

Instrument

Software

Cell population abundance

Gating strategy

Tick this box to confirm that a figure exemplifying the gating strategy is provided in the Supplementary Information.

Magnetic resonance imaging

Experimental design

Design type

Design specifications

Behavioral performance measures

Acquisition

Imaging type(s)

Field strength

Sequence & imaging parameters

Area of acquisition

Diffusion MRI Used ☐ Not used

Preprocessing

Preprocessing software

Normalization

Normalization template

Noise and artifact removal

Volume censoring

modeling & inference

Model type and settings

Effect(s) tested

Specify type of analysis: Whole brain ROI-based Both

Statistic type for inference

(See Eklund et al. 2016)

Correction

Models & analysis

n/a Involved in the study

- Functional and/or effective connectivity
- Graph analysis
- Multivariate modeling or predictive analysis

Functional and/or effective connectivity

Graph analysis

Multivariate modeling and predictive analysis
